# Variation of presence/absence genes among *Arabidopsis* populations

**DOI:** 10.1186/1471-2148-12-86

**Published:** 2012-06-14

**Authors:** Shengjun Tan, Yan Zhong, Huan Hou, Sihai Yang, Dacheng Tian

**Affiliations:** 1 State Key Laboratory of Pharmaceutical Biotechnology, Department of Biology, Nanjing University, Nanjing, 210093, China

## Abstract

**Background:**

Gene presence/absence (P/A) polymorphisms are commonly observed in plants and are important in individual adaptation and species differentiation. Detecting their abundance, distribution and variation among individuals would help to understand the role played by these polymorphisms in a given species. The recently sequenced 80 *Arabidopsis* genomes provide an opportunity to address these questions.

**Results:**

By systematically investigating these accessions, we identified 2,407 P/A genes (or 8.9%) absent in one or more genomes, averaging 444 absent genes per accession. 50.6% of P/A genes belonged to multi-copy gene families, or 31.0% to clustered genes. However, the highest proportion of P/A genes, outnumbered in singleton genes, was observed in the regions near centromeres. In addition, a significant correlation was observed between the P/A gene frequency among the 80 accessions and the diversity level at P/A loci. Furthermore, the proportion of P/A genes was different among functional gene categories. Finally, a P/A gene tree showed a diversified population structure in the worldwide *Arabidopsis* accessions.

**Conclusions:**

An estimate of P/A genes and their frequency distribution in the worldwide *Arabidopsis* accessions was obtained. Our results suggest that there are diverse mechanisms to generate or maintain P/A genes, by which individuals and functionally different genes can selectively maintain P/A polymorphisms for a specific adaptation.

## Background

Sequence variants affecting phenotypes between different individuals were believed to be mostly due to small differences, such as single nucleotide polymorphisms (SNPs) [1-4]. However, when comparing two or more genomes within a species, gene presence/absence (P/A) variations have been also commonly observed in recent studies. Since Grant *et al.* found P/A polymorphisms in the *RPM1* gene in *Arabidopsis*[5], an increasing number of P/A genes have been reported in disease resistance genes in this model species [6,7] and other land plants [8-10]. This phenomenon has also been described in the human genome [11-13], the telomeric region in *Drosophila*[14] as well as bacterial genomes [15], which suggests that P/A polymorphisms have unique roles in species differentiation. Additionally, several human diseases have been associated with gene insertions or deletions [16,17] and in plants, there is evidence that P/A genes are involved in gene expression [18] and noncollinearity in heterosis [19]. These examples indicate the importance of P/A genes in the evolutionary history of various species.

The commonly used definition of a P/A gene is that it is a gene present in some individuals but absent in others within a species at a particular locus, although there are different definitions in the literature [6,10]. The narrow definition of a P/A gene is one which exists only in one individual but not in another on a genome-wide scale. For example, it was reported in maize that 20% of genome segments (~10,000 genes or gene fragments) are not shared between inbred lines B73 and Mo17 [8]. Yu *et al.* found that 2.2% and 3.3% of rice *indica* and *japonica* genes, respectively, are unique to the subspecies [20], while Ding *et al.* found 5.2% genes with P/A polymorphisms between Nipponbare and 93-11 [10]. Although a gene can be localized to a genomic position and be denoted as a P/A gene at that locus, it may have a paralog at a different locus. By using a broad definition, 4.7% additional genes were classified as P/A genes among rice genomes [10]. Our study also uses the broad definition of a P/A gene, which is one being found at a particular locus only in some genomes compared to the others.

Most land plants have evolved by whole genome duplication and subsequent gene loss [21]. Such extensive rearrangement events can result in a high proportion of P/A genes in plants. Transposable elements (TE) are dominant factors inducing intraspecies diversity in maize [8]. Large duplications can be another source of genetic variation [22]. In *Arabidopsis*, unequal and illegitimate recombination also plays an important role in triggering large-scale indels [23]. The *Arabidopsis* genome is extremely redundant due to segmental duplications and tandem arrays [24]. These features provide ample opportunity for unequal crossing over to generate P/A genes. Balancing selection is thought to be one of the mechanisms maintaining P/A polymorphisms, at least for some disease resistance P/A genes [6,7,25]. However, compared with the large numbers of detected P/A polymorphisms, the mechanisms for P/A gene generation and maintenance are complicated and remain unclear.

Although P/A polymorphisms have been reported in several species [6,23], there is still a lack of a clear estimate of the P/A gene number, proportion and variation pattern in any particular species, since a large number of fully sequenced individual genomes is the basic prerequisite for such studies. Recently, 80 re-sequenced *Arabidopsis* genomes were released [26,27] and provided a unique opportunity to systematically study the characteristics of P/A genes. By analyzing the data, we identified a remarkable number of P/A genes and obtained an estimate of the P/A genes and their frequency distribution in the worldwide *Arabidopsis* accessions. We also used this information to investigate the variation in P/A gene patterns among accessions and to provide a description of their preference locations on chromosomes. An analysis of the relationship between diversity and frequency of P/A genes was performed to explore the natural selection pressure, the evolutionary forces on P/A genes in *Arabidopsis* populations as well as the mechanism for P/A generation.

## Results

### Identification of P/A genes

**Figure 1:**
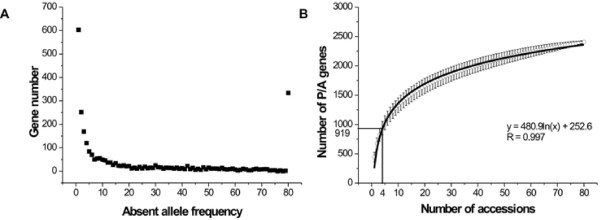
**Distribution of P/A frequencies among 80 Arabidopsis accessions.** (**A**) Number of P/A genes vs. absent allele frequency. (**B**) Expected increase of P/A genes relative to the number of sampled accessions, based on a random sampling model. Each datapoint represents an average number of P/A genes for 10000 random simulation samples, and the 95% confidence intervals were also indicated. For example, when 4 accessions are sampled, 919 absent genes are expected.

Out of the total 27,004 genes in the *Arabidopsis thaliana* Columbia (Col) line, 2741 genes (10%) were found absent in one or more genomes of the 80 *Arabidopsis* accessions using our approach. Among these genes, 603 were absent in only one accession as shown in Figure 1A. As the absent allele frequency in accessions increased, the number of missing genes decreased rapidly, except at the last outlying datapoint on this graph (Figure 1A) which represents 334 genes absent in all 80 accessions. In other words, these genes were present only in the Col genome.

Since a remarkable proportion (10%) of genes were found absent in multiple *Arabidopsis* accessions, it was especially important to determine whether the identified genes were truly P/A loci. Three methods were used to evaluate the reliability of the identification of these genes. First, we built a random model to predict the number of absent genes for a certain number of accessions by using data from the identified P/A genes in the 80 accessions. In this manner, a formula (y = 480.9ln(x) + 252.6) was obtained when up to 80 accessions were randomly sampled (Figure 1B). The trendline in Figure 1B demonstrates that the number of absent genes increases logarithmically when the number of accessions sampled increases linearly, and we could estimate the absent gene number at each datapoint. For example, when 4 accessions are sampled, a total of 919 absent genes are expected based on the formula. Fortunately, there are four independent populations (C24, Bur-0, Kro-0 and Ler-1; [28]) which have been fully sequenced with high genome coverage (50–200×). We used the same methods as for the 80 accessions to identify the P/A genes in these four populations. A consistent result between the number of absent genes identified from independent samples and that predicted by the formula above would help to validate the P/A genes identified in the 80 accessions. In fact, 980 P/A genes were identified in the four independent populations, not significantly deviated from the number (919) predicted by the formula above (from randomization: *P* = 0.77), indicating a general reliability of the P/A genes identified.

Second, we determined whether some of the P/A genes were truly absent by PCR. We randomly chose 53 genes for genotyping in randomly selected accessions. Of the 38 less frequent P/A genes which were absent in one or ten accessions, nine genes (or 23.7%) were determined to be false (Additional file 1: Table S2; Additional file 2: Table S4). Meanwhile among the remaining 15 genes, which had a higher absence frequency, two of them (13.3%) were not true P/A genes. Therefore, 11 out of the 53 P/A genes were not truly absent, while the other 42 were confirmed to be absent (33 out of 42) or did not generate PCR products (9 out of 42), most likely due to the absence of the regions containing the primer sequences. Our PCR results demonstrated that the rate of wrongly inferred P/A genes was 25% (=11/(53−9)) after excluding the uncertain results, while the majority of P/A genes (75%) initially identified were truly absent.

Finally, we evaluated the reliability of the outlier in Figure 1A, the last datapoint with the abnormally high value of 334 P/A genes. This number could be accurate when there are large numbers of population-specific genes. In this case, it was reasonable for Col, which was supported by BAC-clone based genome sequences, to have many P/A genes; however, the other accessions could not have had such high numbers, because the re-sequenced genomes used Col as a reference and therefore would not produce additional accession-specific P/A genes. However, when all 334 genes were subjected to BLAST search against the four genomes of C24, Bur-0, Kro-0 and Ler-1, 198 out of the 334 genes produced no matches, while 8 had alleles in all four genomes. The BLAST results suggested that there were accession-specific P/A genes, but a large proportion (40.7%) of the 334 genes were not reliable, probably because these genes could not be easily re-sequenced in the 80 accessions. Thus, all of the genes at this datapoint were discarded in our subsequent analyses.

Of the remaining 2,407 P/A genes, the average number of absent genes per accession was 444 (ranging from 362 to 555), which meant that 1.64% of the genes were absent compared with those of Col. This proportion was slightly lower than that reported in rice [10,20] and maize [8]. Additionally, nine *Arabidopsis* resistance genes with known P/A polymorphisms [6] were found to be included in these P/A genes, further supporting the validity of the method for their identification in this study.

### Characteristics of P/A genes among populations

We next determined whether the P/A genes were transcriptionally active. Some genes, such as pseudogenes are not expressed, and therefore they would not affect biological processes in an individual. After BLAST searches in the Arabidopsis Transcriptome Genomic Express Database, we found evidence of expression for all of the 2,407 genes, suggesting that the P/A genes have biological functions. However, this analysis was performed based on the Col annotation, and we could not confirm expression of these genes from other accessions.

It is rational to assume that many P/A genes may come from multi-copy gene families, and the homologous genes may have overlapping functions. To better group the homologs, genes with a nucleotide divergence less than 0.3 were defined as a family. By this definition, 1,218 P/A genes (or 50.6%) were grouped into 718 families, but 1,189 singleton genes (or 49.4%) still lacked homologs in the Col genome. In the 24,570 non-P/A genes (27,004 total genes minus the 2,407 P/A genes), there were 8,247 genes (33.5%) which belonged to multi-gene families. There was a significantly higher proportion of multi-genes in P/As than that of non-P/A genes (*χ*^2^ = 281, df = 1), implying that the multi-copy genes were more likely to have P/A polymorphisms.

Large deletions or the absence of genes can be derived from unequal recombination between homologs [23]. Tandemly arranged or clustered genes are also abundant resources for generating gene conversion and unequal crossover [29]. Therefore, the P/A genes may be a consequence of unequal recombination between clustered genes. Here, we classified the P/A genes into clusters to investigate their physical locations (definitions in Methods). In total, 1,272 gene clusters were found in the Col genome, of which 463 clusters contained 746 P/A genes. In other words, 31.0% P/A genes were clustered on chromosomes, significantly higher than the 12.0% of clustered genes in the whole Col genome (*χ*^2^ = 915, df = 1).

### Co-variation between absent allele frequency and nucleotide diversity or gene length

**Figure 2:**
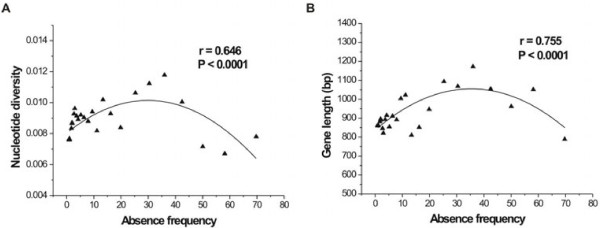
Co-variation between absent allele frequency and nucleotide diversity (A) or gene length (B).

The average nucleotide diversity of all the present alleles at the P/A loci was 0.0088. The level of nucleotide diversity may be associated with the frequency of absent alleles within a population. According to this hypothesis, a high frequency of absent alleles could be maintained with higher probability by balancing selection, an indication of higher diversity at a locus. To detect this association, the 2,407 P/A genes were equally sorted into 29 bins based on their frequencies among accessions, such that each of these bins contained 83 genes. Figure 2A shows the relationship between the frequency of absent alleles and the diversity of present alleles at a P/A locus. The increase in absent allele frequency (represented by the number of absent alleles from 1 to 80 accessions) positively correlated with the average nucleotide diversity until the number of absent alleles reached 40 out of 80 accessions (or ~0.5 frequency of absent alleles), after which the average diversity decreased. Thus, there was a significant quadratic-like correlation between the nucleotide diversity and frequency of absent alleles (r = 0.65; *P* < 0.0001). Additionally, a similar pattern was found when investigating the relationship between the frequency of absent alleles and the length of P/A genes (Figure 2B). The implications of these correlations remain to be clarified.

### Variation of P/A genes among populations

To examine variations in the pattern of P/A genes among accessions, a phylogenetic tree, based on the presence or absence of the 2,407 genes, was constructed. We observed some extent of differentiation in these genes among the 80 accessions based on geographic origin (Additional file 3: Figure S5A). Many genes with similar polymorphic patterns were found in accessions from the same countries. That is, these closely located accessions were likely to have similar spectrums of P/A genes. Some accessions from the same geographic area, such as Bak-2 and Bak-7, had highly similar patterns of P/A genes.

**Figure 3:**
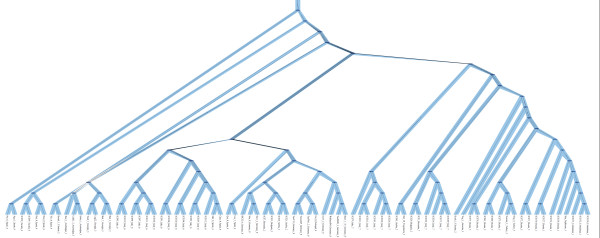
**The reconciled tree.** This tree was viewed as the evolved P/A tree inside the accession tree. The accession tree was shown in blue and the P/A tree in black. At the edges of branches were the accession names, countries and 8 regions where they were sampled. Most of the P/A tree vertices matched the accession tree vertices (dark blue ellipses) particularly in each geographic clade, which meant the P/A polymorphic pattern was associated with individual variation.

To better characterize the geographic distribution of P/A polymorphisms, a reconciled tree (Figure 3) was constructed to view the evolved P/A tree over an accession tree, built by using all SNPs of non-P/A genes (Additional file 3: Figure S5B). In the reconciled tree, we could see that the 80 accessions (shown in blue) were generally separated into eight geographic regions where they were sampled, and the P/A tree (shown in black) matched the accession tree particularly in each of the 8 clades (Figure 3), indicating there was a strong correlation between P/A pattern and individual variation. Both trees (reconciled and P/A trees) demonstrated that P/A polymorphisms were related to the geographic regions, suggesting that population structure may play an important role in the generation/maintenance of P/A genes.

However, each branch (or accession) was quite different from others in the P/A gene tree. It was especially remarkable that no identical branches could be detected among the 80 accessions. The closest accessions were ICE169 and ICE173 (Additional file 4: Table S1), which had no SNP detected in the non-P/A genes between them (Additional file 3: Figure S5A), but they still had different P/A polymorphic patterns at 26 loci. The same phenomena were found in comparisons between ICE150 and ICE152 as well as between ICE212 and ICE 213.

### Distribution of P/A genes on chromosomes

**Figure 4:**
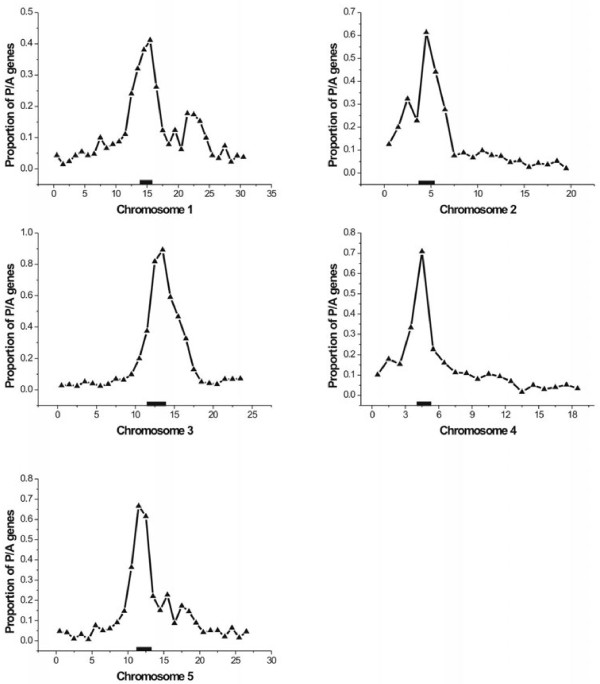
**Distributions of P/A genes on chromosomes.** The x-axis represents the physical distance (Mb) on the chromosome. The y-axis represents the proportion of P/A genes for each Mb of chromosomal length. The thick black bar represents the centromeric region.

The five chromosomes were divided into 1 Mb sections and centromere positions were established as previously reported [30]. Interestingly, a markedly nonrandom distribution of P/A genes was revealed along the chromosomes (Figure 4). The proportion of P/A genes (the number of P/A genes divided by number of all genes in each section) was always the highest near the centromere, and decreased monotonically to background levels over several megabases. This phenomenon could be attributed to the low gene density in the areas around centromeres, and thus the gene-rich regions were expected to have more P/A genes but not higher proportions. However, detailed examination showed the opposite finding. For example on chromosome 3, the highest gene-density section with a total of 329 genes contained only nine P/A genes. Meanwhile there were 27 P/A genes in the centromeric section where there were a total of 33 genes. The same pattern was also observed on chromosome 5 (5:307 and 12:18, respectively, for P/A to total genes) and other chromosomes (Additional file 5: Figure S1; Additional file 6: Table S3).

### Functional categories of P/A genes

Gene Ontology (GO) terms are widely used as the standard for functional annotation. The 22,570 *Arabidopsis* genes have been assigned at least one GO annotation from the TAIR and The Institute of Genomic Research (TIGR) databases [31]. The three organizing principles of GO are cellular component, molecular function and biological process [32], and according to the ‘GO Slim’ categories, genes are classified into small groups within each GO hierarchy.

Of the cellular component genes, 738 P/A genes (65.9%) were related to membranes, comprising the largest proportion besides unknown components (Additional file 7: Figure S3). Compared to the overall frequency of membrane genes (37.5%, [31]), the membrane-related P/A genes were significantly overrepresented (*χ*^2^ = 359, df = 1). Most of them encoded proteins in the endomembrane system (555 P/As), and the others were distributed within the plasma, chloroplast and Golgi membranes. When analyzing the molecular functions, 888 P/A genes (48.8%) encoded proteins with binding functions, mostly binding with protein and nucleotides, and were also overrepresented (*χ*^2^ = 167, df = 1). However, 121 encoded proteins in the ‘other binding’ category could bind to zinc ion, while the remaining proteins had various ion binding functions. The activity terms of many enzymes, including hydrolases, transferases and kinases, were also numerous in the molecular function category. In the category of biological process, the highest proportion of these were related to stress responses (23.8% vs 2.2%, *χ*^2^ = 1466, df = 1).

**Figure 5:**
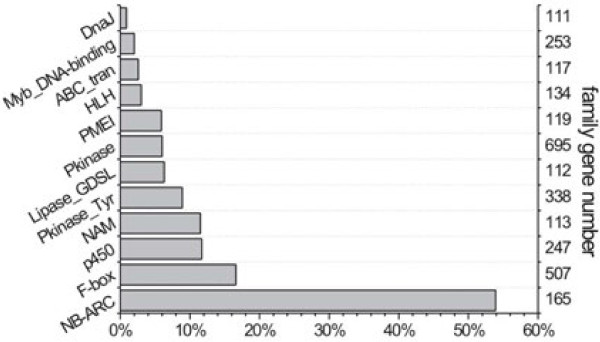
**Distribution of P/A genes in different functional categories.** The y-axis on the right is the number of genes in each category and the x-axis is the proportion of P/A genes.

Moreover, we analyzed the frequency of P/A genes in different gene classes identified by Pfam domain database [33]. In *Arabidopsis*, 75% ~ 82% functional domains could be predicted by Pfam [34,35], and such a high proportion could cover most of the fundamental gene classes. Classes involved in basic biological processes (such as heat shock protein and ABC transporter) and in regulation of transcription (such as Myb and HLH) had less impact (see Figure 5). By contrast, disease resistance genes and F-box gene classes were affected extensively (53.9% and 16.6%, respectively). In NBS proteins, the LRR domains are thought to mediate interactions with pathogen-derived molecules and reported to be highly variable [36]. This finding is consistent with previous studies in which the NBS genes often show P/A polymorphisms in *Arabidopsis* and other plants [5,6,10,37]. Additionally, F-box genes are thought to have undergone rapid birth and death in the *Arabidopsis* genome [38], as well as evolved quickly in response to pathogen pressure [39].

## Discussion

### Number of P/A genes among populations

Using our approach, 2,407 genes were identified with P/A polymorphisms, indicating that about 10% of genes are likely to be maintained as P/A genes in *Arabidopsis*. Both the number and the proportion of P/A genes are remarkable, which may reflect their important roles in the species adaptation to the environment.

However, our approach could have overestimated the number of P/A genes. The PCR results indicated that 25% of the P/A genes were falsely identified. On the other hand, the number identified could be underestimated, because the proportion of false P/A genes was predicted to be much lower when the absent frequency was higher. Moreover, only P/A genes which are in deletions, compared with the Col genome, could be identified, based on the limitation of re-sequencing methods for the 80 genomes. If the P/A genes in insertions could be identified, the number of P/A genes could double. In fact, most of the 334 P/A genes present only in Col but absent in all 80 accessions should represent P/A genes in Col insertions, relative to the 80 accessions. This number was similar to the average number (444) of P/A genes per accession. In this case, each accession was expected to contain 2.47% P/A genes. Although the expected proportion is likely higher by two-fold, it was lower than that in rice (5.2%) and maize (~20%) for each individual.

The difference in proportion of P/A genes identified here in *Arabidopsis*, compared with rice and maize, could be due to the genome sizes, methods for pollination, transposon activity and/or domestication. The maize genome undergoes recurrent transposon events; therefore, observation of widespread DNA sequence noncollinearity would not be unexpected [8]. The outcrossing method and the large genome size may also contribute to the high proportion of P/A genes. The rice genome is three times larger than that of the *A. thaliana* genome, which is more compact consisting largely of unique sequences [38]. Moreover, the previous comparison of rice genomes was based on two individual plants under high pressure of artificial selection in different regions [20], which would genetically create higher divergence [40]. These characteristics may explain the greater number of P/A genes observed in rice.

Previously, Clark *et al.* found that 2,495, or 9.4%, of *A. thaliana* protein coding genes were affected by large effect changes when comparing 19 accessions and one reference, Col [38]. Meanwhile, according to our formula, there should be 1,669 absent genes when 19 accessions were randomly sampled. This number was less than the 2,495 indicated by the authors above because the large effect changes included indels as well as SNPs and highly dissimilar sequences. Ziolkowski *et al.* found large indels (>100 bp) distributed within 908 CDS regions by comparing genomic data from two *A. thaliana* lines, Col and Ler [23]. This number was also larger than ours since not all indels result in gene deletions.

On the other hand, pseudogenization could be considered as another way of gene loss. Across the 80 accessions, Cao *et al.* found a total of 6,197 genes with 12,468 SNPs that caused large effects on gene structure, for example altered start codons, introduced premature stop codons, or affected splice donor or acceptor sites, were found [26]. Consistent with their results, we found 7,602 genes contained frameshift mutations or premature stop codons affected by both SNPs and indels in these accessions. The numerous P/A and pseudo- genes demonstrated that there were frequent gene loss events in *Arabidopsis* populations. Most interestingly, P/A and pseudo- genes had similar distribution in gene categories and on chromosomes. For example, there were a high proportion of pseudo- or P/A genes in NBS, F-box and p450 gene families. These results suggested that both pseudo- and P/A polymorphisms may play similar roles in contributing to the dynamic variations in some functional loci, e.g. through pseudogenization and P/A to escape the fitness costs [37].

### Mechanisms of P/A gene distribution on chromosomes

Our results revealed that the distribution of P/A genes was not random on chromosomes and that their proportion was especially high around centromeres. The P/A genes could be easily generated by unequal recombination between homologs, especially tandemly arranged genes. In this scenario, a higher proportion was expected in the areas with dense gene clusters. Previous studies [23,29] indeed support this mechanism to engender P/A polymorphisms in *A. thaliana*. Generally, *A. thaliana* has a relatively compact genome and contains many unique sequences, e.g., 64.5% singletons by our definition in which 6.78% of P/A genes were identified. Nevertheless, there was a significantly higher proportion of P/A genes (50.6%) found in the multi-gene families. Within this multi-gene group with P/A genes, a significantly higher percentage of clustered P/A genes (746/1,218 = 61.2%) was observed. Although with some variation, the proportion of clustered P/A genes was indeed much higher on the chromosome arms (Additional file 8: Figure S2) as expected. Meanwhile on chromosomes 1 and 5, there were several P/A secondary peaks (Figure 4), at which most of the clustered genes were located (Additional file 8: Figure S2; Additional file 6: Table S3).

However, a relatively smaller number of P/A genes clustered near the centromeric regions (Additional file 8: Figure S2). This finding suggested that the unequal recombination events among P/A genes were not frequent in these regions, which is also reasonable since centromeres are reported to have low recombination frequencies [41]. However, we observed a high proportion of P/A genes (the number of P/A genes divided by number of all genes) near centromeres, indicating that other mechanisms generated P/A genes in these areas. Centromeres have highly conserved functions, serving as the site for kinetochore formation and sister chromatid cohesion, while it is also the most dynamic chromosomal region [42,43]. Although the explanation for rapid evolutionary dynamics is still under debate [44], stochastic events such as mutation and genetic exchange are apparent [45]. Widespread gene conversion has also been observed in these regions [46]. Unlike chromosome arms which have high gene density, the centromere usually contains specific types of DNA sequences that are typically tandem repetitive sequences [41], and its gene density is much lower in higher eukaryotes. Therefore, the characteristics in these areas could induce a high rate of sequence exchanges, which may result in P/A polymorphisms. With a similar evolutionary characterization to the centromere, the telomeric region also contains much repetitive sequences. In Drosophila, Kern found a number of duplication and deletion polymorphisms in this region [14]. However, we did not detect a high P/A proportion in *Arabidopsis* (Figure 4), which could be resulted from the evolutionary and genetic differences between plants and insects.

The generation of P/A genes in centromeric regions is clearly different from other areas. In the less repetitive regions, gene clusters could add or delete copies, which eventually could produce P/A genes. Meanwhile in a region with abundant repeats, the unequal crossover among repeat sequences could cause the non-clustered genes to be added or lost, particularly singleton genes on chromosome arms and near the centromeres. It seems that there always are efficient ways to engender P/A polymorphisms in different regions.

In addition, *Arabidopsis* underwent a paleopolyploidy event before splitting from *Brassica*, which could produce abundant homologous genes and repeat sequences. Although gene duplication is the major mechanism of sub- and neo-functionalization for a species to adapt to the new environment [47], the redundant genes are great burdens for a host. Therefore, the gene loss, which includes deletion and pseudogenization, could be an effective strategy to economize the cost. This explanation may primarily account for the higher P/A polymorphisms among homologous genes.

### Evolutionary forces for P/A genes in groups and populations

Based on the GO analysis, the proportion of P/A genes were quite different among gene function categories. Within the GO cellular component group, the proportions of P/A genes related to membrane and binding were found to be much higher than the others, although these two classes of genes are also relatively high within the whole *Arabidopsis* genome [31]. Most interestingly, in the category of biological process, the ‘response to stress’ group comprised the highest proportion of P/A genes (23.8%, Additional file 7: Figure S3), while its portion is only 2.4% of the whole *Arabidopsis* genome after excluding the biological process unknown genes. In this group, more than half were disease resistance genes. Similar results were observed in the gene class analysis based on Pfam domain database [33] that revealed a high proportion of P/A genes in the NBS gene class (53.9%, Figure 5). The varied distribution of P/A genes in different gene groups suggested that their proportions developed under natural selection. Some NBS genes were reported to have P/A polymorphisms as they have hypersensitive responses to an avirulence protein encoded by a pathogen in *Arabidopsis*[6,37]. The co-evolution between the host and pathogen naturally leads to variable characteristics of NBS genes adapting to rapidly changing pathogenic populations. By contrast, classes involved in basic biological processes would contain fewer P/A genes due to their conserved functions.

In this study, an unknown evolutionary force was also indicated in the distribution of P/A genes among accessions, which exhibited little geographic differentiation based on the highly diversified branches in the P/A gene tree (Additional file 3: S5A). The fact that there were no identical branches detected indicated that the P/A genes occurred commonly and maintained selectively in the worldwide *Arabidopsis* populations. For example, the two closest accessions ICE169 and ICE173 (Additional file 4: Table S1) still had different P/A polymorphic patterns at 26 loci (Additional file 3: Figure S5A). Both accessions within this pair were in similar environments, but their P/A patterns were relatively dissimilar. It seems a balance exists to maintain diverse sets of P/A genes, beyond the limitation of the geography-specific factors, biotic or abiotic, to enforce the fixation of P/A genes at a location.

### Nucleotide diversity of presence alleles at P/A loci

In our study, we observed a pattern of co-variation between absent allele frequency and nucleotide diversity among populations (Figure 2). To obtain convincing evidence of such a relationship, genes within the same absent frequency were randomly sorted, and then the 2,407 P/A genes were equally sorted into 29 bins of 83 genes each based on their frequencies among accessions. A figure was also plotted based on the average nucleotide diversity under each frequency (Additional file 9: Figure S4A). In these different analyses, a significant co-variation was still observed between the values of the nucleotide diversity and their frequencies.

When the absent accession number was about 40, which also meant the P/A frequency was at the highest (P: A = 40: 40), the highest average diversity was detected within present alleles. When the P/A frequency was low, the nucleotide diversity was also lower. A high level of diversity at a locus indicates that the polymorphism has been maintained for a longer time. That a higher frequency of a P/A gene equates with an older time of origin suggests that this type of polymorphism is relatively stably maintained, probably by natural selection. Balancing selection may be one of these selective forces, which for example was suggested to act on the *RPS5* gene (AT1G12220) [25], with a high absent frequency of 32/80 and a high nucleotide diversity of 0.0136. Interestingly, the signature of balancing selection at this locus was similar to the patterns observed in this study. This example suggests that balancing selection favors guarding resistance proteins based on the co-evolutionary arms race model [48]. Indeed, when we compared the P/A gene numbers between different functional categories, 50.6% of disease resistance associated genes had a relatively high absent frequency (20–60 out of 80 accessions). This proportion was much higher than those in other groups, such as F-box (16.7%) or p450 (31.0%). The highest proportions of both P/A genes and absent frequency indicate that some disease resistance related genes are more likely to be under balancing selection to respond to variations in corresponding pathogens. By contrast, a lower diversity means that the gene is relatively young, and therefore its P/A frequency is lower and probably subject to random drifting. The younger or recently generated P/A genes may be in flux, and their fate may either be to eventually become deleted or fixed by random drifting or natural selection. These types of P/A genes are more likely to be under neutral mutational and population genetic processes.

## Conclusions

We identified a remarkable number of P/A genes in the worldwide *Arabidopsis* populations, which reflected their important roles in individual adaptation. The distribution of P/A genes was uneven on chromosomes and 31.0% of them were clustered, suggesting that unequal recombination was a major mechanism for generating P/A polymorphisms. However, a high proportion of P/A genes were observed near the centromeres, which meant the generation of P/A genes was different in these regions. We also observed a varied distribution of P/A genes in different gene groups, which indicated they were under natural selection. Most importantly, little geographic differentiation was found in the distribution of P/A genes among populations, implying that these genes were generated and maintained randomly at relatively high frequency. Additionally, we observed a relationship between diversity and frequency of P/A genes. The high nucleotide diversity and high absent gene frequency suggested that balancing selection could be a mechanism maintaining P/A polymorphisms. This work may help us to better understand the evolutionary forces on P/A genes in *Arabidopsis* populations as well as the mechanisms for P/A generation.

## Methods

### Databases

The genome of *Arabidopsis* ecotype Columbia (Col) was downloaded from The Arabidopsis Information Resource (TAIR) database (http://www.arabidopsis.org/). The sequences of 80 *A. thaliana* accessions (10 ~ 24× coverage) were downloaded from the 1001 Genomes Data Center (http://1001genomes.org; [26]). This data center only provides files describing the position-wise bases of 80 accessions against the Col assembly. Therefore, we assembled the whole genome sequences of each accession according to the TAIR8 annotated positions. Subsequently, the protein coding regions (CDS) of each accession were obtained based on the annotation of TAIR8, while the CDS with ambiguous bases were eliminated. For multi-transcript genes, only the longest CDS was chosen to represent each of those genes. The TE genes were also excluded. All calculations were implemented using Perl scripts.

### Identification of absent genes and construction of trees

Some CDS of certain accessions contained ‘inaccessible regions’ defined by the 1001 Genomes Data Center. Such inaccessible regions were either large deletions or lacked base calling in re-sequencing analysis. If the length of these regions were more than half of the CDS length, we defined this gene as an absence in this accession. Since some of the absent genes may be caused by sequencing error or misassembly, we evaluated the reliability of the P/A genes by examining the other sequenced genomes. In the data center, there were four assembled *A. thaliana* accessions, C24, Bur-0, Kro-0 and Ler-1 [28]. These four lines were not included in the 80 accessions, and instead were used as reference genomes to validate the P/A genes. The predicted genes were examined by BLASTN (e value <10^−10^) search in the local database. If one gene had a sequence counterpart which was >50% in length, it was determined to be present in this accession.

All of the candidate genes were subjected to BLASTN search for expression analysis. Expression support was given to the 26,541 annotated *A. thaliana* coding genes on the basis of full-length cDNAs, expressed sequence tags (ESTs), massively parallel signature sequencing (MPSS) and genome-wide tiling array transcriptome (http://signal.salk.edu/cgi-bin/atta; [49]).

To group the *A. thaliana* accessions with respect to the pattern of P/A genes, a phylogenetic tree was created by using P/A polymorphisms among accessions, based on neighbor-joining method with p-distance model by PAUP* 4.0 [50]. In addition, an accession tree was constructed by using all SNPs of non-P/A genes among genomes, based on the Neighbor-joining method [51]. A reconciled tree, which was viewed as the evolved P/A tree in the accession tree, was then illustrated using primetv software [52].

### Classification of multi-copy gene families, gene clusters and functional categories

We built a local database of all Col genes, and then every gene was subjected to the BLASTN search to identify homologous genes. A criterion of ≥70% nucleotide identity was used to define homologs in multi-copy gene families [10]. Otherwise, genes were categorized as singletons.

A gene cluster was defined as a region that contained two or more homologs which were close to each other (≤ eight genes between any two of them, [53]). All absent genes were searched whether they resided together in a gene cluster or not. Subsequently, these genes were subjected to searches of the Gene Ontology (GO) annotated database for functional categorization (http://www.arabidopsis.org/tools/bulk/go/index.jsp) and of the Pfam database (Pfam 24.0, [33]) to identify the functional domains of the genes.

### Verification of absent genes by genotyping

To confirm the reliability of P/A genes identified, 53 of them were randomly selected for genotyping by PCR. The seeds of 80 *Arabidopsis* accessions (Additional file 4: Table S1) were purchased from the 1001 Genomes Data Center. Twenty-four lines were randomly chosen to verify the absent genes, with Col as the positive control. For each gene, at least eight lines were chosen to perform a three-primer PCR amplification (two primers were designed in the 3′- and 5′-flanking regions of the breaking point and one in the insertion of the P/A gene), from which, the present or absent genotype was expected to yield alternative products.

## Competing interests

The authors declare that they have no competing interests.

## Authors’ contributions

DT, SY and ST designed the study. ST contributed extensively to the bioinformatic analyses. ST and YZ performed PCR experiments and HH assisted the analyses. DT, ST and SY wrote the manuscript. DT and ST prepared and revised the manuscript. All authors read and approved the final manuscript.

## Supplementary Material

Additional file 1 **Table S2.**The genotyping results. Click here for file

Additional file 2 **Table S4.**Primer information for the PCR amplification of P/A genes in Table S2. Click here for file

Additional file 3 **Figure S5.**The P/A tree and accession tree. Click here for file

Additional file 4 **Table S1.**The 80 accessions used in this study. Click here for file

Additional file 5 **Figure S1.**Distributions of P/A genes on chromosomes. Click here for file

Additional file 6 **Table S3.**Distributions of P/A genes on chromosomes. Click here for file

Additional file 7 **Figure S3.**GO categories of P/A genes. Click here for file

Additional file 8 **Figure S2.**Distributions of clustered P/A genes on chromosomes. Click here for file

Additional file 9 **Figure S4.**Co-variation between absent allele frequency and nucleotide diversity or gene length. Click here for file
