# Supplementary material for: Variation of presence/absence genes among Arabidopsis populations
Source: BMC Evol Biol. 2012 Jun 14;12:86. doi: 10.1186/1471-2148-12-86 (PMC3433342; doi:10.1186/1471-2148-12-86)
Supplement: Additional file 1 — Table S2.The genotyping results. [file 1471-2148-12-86-S1.pdf]

Table S2. The genotyping results.

Gene name	Na	Col	Agu-1	Bak-2	Bak-7	Dog-4	Fei-0	HKT24	ICE120	ICE153	ICE163	ICE169	ICE21	ICE212	Kastel-1	Koch-1	Lag22	Lerik1-3	Mer-6	Nemru	Ped-0	Sha	Tuescha	WalhaesB	Xan-1	Yeg-1	Validation
AT1G07470	1	1/1	1/1	-	-	1/1	1/1	1/1	-	-	-	-	-	-	-	-	0/1	-	-	1/1	1/1	-	-	1/1	-	-	F
AT1G34650	1	1/1	-	-	-	1/1	1/1	1/1	-	-	-	-	-	-	1/1	1/1	0/0	-	-	-	-	-	1/1	-	-	-	T
AT1G65250	1	1/1	-	-	-	1/1	1/1	-	1/1	1/1	-	-	-	-	-	-	0/1	-	-	-	1/1	-	-	-	1/1	1/1	F
AT2G16810	1	1/1	1/1	1/1	1/1	1/1	-	-	-	-	1/1	1/1	-	-	-	-	-	-	-	-	0/0	-	-	-	-	1/1	T
AT2G19550	1	1/1	-	-	-	1/1	1/1	1/1	-	-	-	-	-	-	1/1	0/0	1/1	-	-	-	-	-	1/1	-	-	-	T
AT2G43930	1	1/1	-	-	-	1/1	1/1	-	0/1	1/1	-	-	-	-	-	-	1/1	-	-	-	1/1	-	-	-	1/1	1/1	F
AT2G45930	1	1/1	-	1/1	-	1/1	1/1	1/1	-	-	1/1	-	-	-	-	-	1/1	-	-	-	-	-	1/1	-	-	0/0	T
AT3G04854	1	1/1	1/1	0/0	-	-	-	-	-	-	-	-	1/1	-	-	-	-	1/1	1/1	-	1/1	-	-	1/1	-	-	T
AT3G05155	1	1/1	1/1	1/1	1/1	1/1	-	-	-	-	0/1	1/1	-	-	-	-	-	-	-	-	1/1	-	-	-	-	1/1	F
AT3G47090	1	1/1	-	1/1	-	1/1	1/1	1/1	-	1/1	-	-	-	-	0/1	1/1	1/1	-	-	-	1/1	-	1/1	-	-	-	F
AT3G59190	1	1/1	-	1/1	-	1/1	1/1	1/1	-	-	0/0	-	-	-	-	-	1/1	-	-	-	-	-	1/1	-	-	1/1	T
AT4G02850	1	1/1	1/1	-	-	1/1	1/1	1/1	-	-	-	-	-	-	-	-	1/1	-	-	1/1	0/0	-	-	1/1	-	-	T
AT4G24974	1	1/1	1/1	1/1	1/1	-	-	-	1/1	-	1/1	-	-	1/1	-	1/1	-	0/0	-	1/1	-	1/1	1/1	1/1	-	1/1	T
AT5G17720	1	1/1	-	1/1	-	1/1	0/0	1/1	-	-	1/1	-	-	-	-	-	1/1	-	-	-	-	-	1/1	-	-	1/1	T
AT5G37520	1	1/1	0/0	1/1	1/1	1/1	-	-	-	-	1/1	1/1	-	-	-	-	-	-	-	-	1/1	-	-	-	-	1/1	T
AT5G53670	1	1/1	1/1	-	-	1/1	1/1	1/1	-	-	-	-	-	-	-	-	1/1	-	-	1/1	0/0	-	-	1/1	-	-	T
AT5G56640	1	1/1	1/1	-	-	-	-	-	1/1	-	1/1	-	-	-	-	0/0	-	-	-	-	-	-	1/1	1/1	-	1/1	T
AT1G03940	2	1/1	-	1/1	1/1	0/1	-	1/1	-	-	-	-	-	-	-	1/1	-	1/1	-	0/1	-	-	-	-	-	-	F
AT1G21870	2	1/1	-	0/0	0/0	-	-	1/1	-	-	1/1	-	-	-	-	1/1	-	1/1	1/1	1/1	-	1/1	-	-	-	-	T
AT1G80700	2	1/1	-	-	0/1	1/1	-	-	1/1	-	-	-	-	-	-	-	1/1	1/1	-	0/1	-	-	-	-	1/1	-	F
AT3G25880	2	1/1	-	-	1/1	1/1	-	-	0/0	-	-	-	-	-	-	-	0/0	1/1	-	1/1	-	-	-	-	1/1	-	T
AT4G10060	2	1/1	-	-	1/1	1/1	-	-	1/1	-	-	-	-	-	-	-	1//1	0/0	-	1/1	-	-	-	-	0/0	-	T
AT4G35165	2	1/1	-	-	1/1	-	-	1/1	-	-	-	-	-	-	-	-	1/1	1/1	-	1/1	0/0	-	1/1	-	0/0	-	T
AT5G42350	2	1/1	-	1/1	1/1	-	-	1/1	-	-	-	-	-	-	-	1/1	-	1/1	0/1	-	0/1	-	1/1	-	-	-	F
AT5G48175	2	1/1	-	1/1	1/1	-	-	1/1	-	-	0/1	-	-	-	-	-	-	1/1	-	-	-	-	1/1	1/1	-	-	F
AT2G23985	3	1/1	0/0	1/1	0/0	-	1/1	-	-	-	-	-	-	-	-	-	-	-	0/0	-	-	-	1/1	-	1/1	-	T
AT2G01360	4	1/1	-	-	0/0	-	-	-	1/1	1/1	-	1/1	1/1	1/1	1/1	1/1	-	1/1	1/1	-	-	-	1/1	1/1	0/0	-	T
AT4G29710	4	1/1	-	1/1	1/1	-	-	-	1/1	-	-	-	-	-	-	-	1/1	1/1	1/1	1/1	1/1	1/1	0/0	1/1	1/1	1/1	T
AT5G65050	4	1/1	-	-	-	-	-	-	1/1	-	-	-	-	-	1/1	-	-	1/1	1/1	1/1	0/0	-	-	-	1/1	-	T
AT4G06490	5	1/1	1/1	-	-	-	-	-	-	1/1	1/1	-	1/1	1/1	0/0	1/1	-	-	-	-	1/1	1/1	-	1/1	-	1/1	T
AT5G38670	6	1/1	-	-	1/1	-	1/1	-	-	-	1/1	-	1/1	1/1	1/1	-	1/1	1/1	-	1/1	-	0/0	1/1	1/1	1/1	1/1	T
AT1G52440	13	1/1	-	-	-	-	-	-	1/1	0/0	-	1/1	-	0/0	-	-	1/1	1/1	1/1	1/1	1/1	0/0	1/1	0/0	1/1	-	T
AT2G29920	15	1/1	-	1/1	1/1	-	-	1/1	1/1	1/1	-	0/0	1/1	0/0	-	-	-	1/1	0/0	-	-	-	-	-	0/0	-	T
AT5G26912	15	1/1	-	-	1/1	-	1/1	1/1	-	1/1	-	1/1	0/0	-	1/1	0/0	-	-	1/1	-	-	-	0/0	-	1/1	-	T
AT2G28426	18	1/1	1/1	-	1/1	-	1/1	0/1	-	1/1	-	1/1	-	1/1	-	1/1	1/1	1/1	-	N	-	0/1	-	-	-	0/1	F
AT4G38280	19	1/1	-	-	-	0/0	-	-	0/0	-	-	-	-	-	0/0	-	0/0	-	-	-	-	1/1	-	1/1	1/1	-	T
AT5G18880	26	1/1	0/0	-	0/0	-	0/0	0/0	-	1/1	-	0/0	1/1	1/1	-	1/1	0/0	1/1	1/0	-	0/0	-	-	0/0	1/1	-	T
AT1G02590	31	1/1	0/0	-	-	-	-	-	1/1	0/0	-	-	1/1	1/1	-	1/1	-	1/1	1/1	0/0	0/0	-	-	-	-	-	T
AT3G11060	31	1/1	0/0	1/1	1/1	-	-	-	0/0	-	-	-	1/1	-	-	-	1/1	-	-	1/1	-	1/1	0/0	-	1/1	0/0	T
AT2G24617	36	1/1	-	-	-	-	-	-	-	0/0	-	-	-	-	0/0	-	-	0/0	0/0	-	-	0/0	0/0	0/0	-	-	T
AT5G18010	40	1/1	0/0	-	-	0/0	-	-	0/0	-	-	0/0	-	-	-	-	-	-	-	0/0	-	1/1	-	-	-	1/1	T
AT5G26270	48	1/1	-	-	0/0	-	0/0	0/0	1/1	-	-	-	-	-	0/0	0/0	-	-	-	-	0/0	-	0/0	0/0	-	0/0	T
AT5G26950	56	1/1	-	-	-	-	1/1	-	-	1/1	-	-	0/0	-	1/0	-	-	-	-	1/1	0/0	-	0/0	-	-	0/0	T
AT1G59920	63	1/1	-	-	0/1	-	0/1	0/1	-	0/1	-	0/1	1/1	1/1	-	0/1	0/1	-	-	-	-	0/1	1/1	-	0/1	0/1	F
AT1G43680	1	1/1	-	1/1	1/1	1/1	1/1	1/1	1/1	1/1	-	-	-	-	1/1	-	1/1	-	-	-	N	-	-	-	1/1	1/1	U
AT2G34320	1	1/1	-	1/1	1/1	1/1	1/1	1/1	1/1	N	-	-	-	-	1/1	-	1/1	-	-	-	1/1	-	-	-	1/1	1/1	U
AT5G56690	1	1/1	-	1/1	1/1	1/1	1/1	N	-	1/1	1/1	-	-	-	-	-	1/1	-	-	-	1/1	-	1/1	-	-	1/1	U
AT1G05740	2	1/1	-	1/1	1/1	-	1/1	-	1/1	-	1/1	-	-	-	1/1	-	-	1/1	1/1	1/1	N	1/1	-	-	1/1	-	U
AT1G47497	2	1/1	-	N	N	1/1	-	1/1	-	-	-	-	-	-	-	1/1	1/1	1/1	-	1/1	-	-	-	-	-	-	U
AT4G18540	2	1/1	-	N	N	-	-	1/1	-	-	-	-	-	-	-	-	1/1	1/1	-	-	1/1	-	1/1	1/1	-	-	U
AT4G01535	10	1/1	-	-	1/1	-	N	-	-	1/1	1/1	1/1	-	-	1/1	-	1/1	-	-	-	N	-	-	1/1	-	1/1	U
AT1G48390	11	1/1	-	1/1	1/1	-	-	-	-	N	-	-	1/1	-	1/1	-	1/1	1/1	1/1	-	-	N	1/1	-	1/1	1/1	U
AT4G09430	20	1/1	-	-	-	-	-	N	-	-	-	1/1	1/1	-	-	1/1	N	1/1	-	1/1	N	-	N	N	-	-	U

Na is the number of absent accessions. The number (1 or 0) before '/' represents the expected P/A pattern, while the one after '/' represents the observed results. '1' means presence and '0' means absence. 'N' means no product and '-' means have not been genotyped. In the 'Validation' column, 'T' is short for true, 'F' is false, and 'U' is 'uncertain' because the absent genes have no genotyping products.
