# Supplementary material for: Variation of presence/absence genes among Arabidopsis populations
Source: BMC Evol Biol. 2012 Jun 14;12:86. doi: 10.1186/1471-2148-12-86 (PMC3433342; doi:10.1186/1471-2148-12-86)
Supplement: Additional file 2 — Table S4.Primer information for the PCR amplification of P/A genes in Table S2. [file 1471-2148-12-86-S2.pdf]

Table S4. Primer information for the PCR amplification of P/A genes in Table S2.

Gene name	primers (5'-3')	Forward or reverse	Length of P contig	Length of A contig
AT1G07470	AATGGATCAGGTGAAGTCAA	F		
	TGGATGGAACTCACAAACTT	R	~1900bp	~1000bp
	GAACTCCTCACGAACTTTGT	R	~500bp	
AT1G34650	CCGCCATCACTCTAATCA	F		
	CGTTTGCATCAGTCTTCA	R	~5400bp	~2600bp
	ACGGGTCAAGTTTGTTCA	F	~1200bp	
AT1G65250	TCTCGAATCCTTTGCTCA	F		
	AGGCTACTCGGATGTTGT	R	~1800bp	~570bp
	TCCACCAGCCAACATCTT	R	~300bp	
AT2G16810	TCTTACCATTCACTCGGA	F		
	GACCGTCATCACTCTTCA	R	~1000bp	~500bp
	CCCACCACTAGACAACTT	R	~400bp	
AT2G19550	CTACCAGCCTGAAAGACA	F		
	ATGGATCTGGACGAGGTT	R	~1800bp	~900bp
	ACCAAGACTGCGATTGTT	R	~400bp	
AT2G43930	TCAGGAGCAAGTTCGTTA	F		
	TGATGTGGCACCGTTAGT	R	~2500bp	~1300bp
	TCAGGCCGAGTTTGTTAT	F	~400bp	
AT2G45930	AATGAGCTTGCCTTGACA	F		
	CCACCAGCCTCGACTTAA	R	~1400bp	~300bp
	AGGATTTGAGTTGCGGTT	R	~700bp	
AT3G04190	GTATACCGGAGAGCTCGTGA	F		
	GCAACATCGCTTAATCCACA	R	~1300bp	~800bp
	AATAGGTAATGCGGTTTTCG	R	~600bp	
AT3G04854	ACTATGCCGAGCTTTGAA	F		
	ATGCTTCCTATGGCTACA	R	~1400bp	~1000bp
	CCTCGTAGTCTCCGGTTA	F	~700bp	
AT3G05155	AGGTCAACCCGTCTATAT	F		
	CGTTCCACAAGTATCAGA	R	~2400bp	~900bp
	GAGTGCAGGATTAGGTTA	F	~360bp	
AT3G47090	CGTCTGGAGCAATGCCTT	F		
	CAGCAGCTTTGAACGTGT	R	~2900bp	~1100bp
	CATGCAAATCCCAACCCT	R	~700bp	
AT3G59190	CGATTGCATGAACCCTAA	F		
	CGTAATGGAGTCCCTGAA	R	~1600bp	~490bp
	GATGGGAGTATGCAATGT	F	~750bp	
AT4G02850	TGCCAAACATACATCACTCA	F		
	TGCTTGCTTCCACTTGATTA	R	~1700bp	~1000bp
	TCAACAGACCAAATCATCGT	R	~790bp	

AT4G24974	ACGGCAACTGAATCACTT	F		
	GGTTTAGCATCCCATCTT	R	~1500bp	~600bp
	CGGAAAATATCTACCCAA	F	~100bp	
AT5G17720	TCCTCCCTCGTAAACTGA	F		
	TTGACAATCTTGGCGTTA	R	~3100bp	~300bp
	GTGCTTACAACATGCGAT	R	~1000bp	
AT5G37520	ATCTCCGTGCAGTCATCT	F		
	AGGCATTTGTCAAGACCA	R	~2300bp	~900bp
	TATGAAACCGAGGGAGTA	F	~250bp	
AT5G53670	GTGCCTCTACGATATGGTCT	F		
	GAAAGTCGAACAATCTGAGC	R	~1600bp	~600bp
	GAAAAGGATAGCGTCAGATA	R	~300bp	
AT5G56640	GGCTTAACCTTCTCGACA	F		
	TCGTCTCCTAAACCATCA	R	~2000bp	~600bp
	TAGCATTGCACAAGGCTG	R	~200bp	
AT1G03940	TACCCGCGCTTCTGAGAT	F		
	TTGGCAGGTATGGTATCC	R	~2600bp	~1300bp
	GACGCCATGCAACACACT	F	~950bp	
AT1G21870	GCATTGCCACATTCCTAA	F		
	GCGGTAAATTCAGCAACA	R	~2100bp	~1000bp
	TCCAACAATAGGCACCAA	R	~600bp	
AT1G80700	CGCTATCTGATCGAACCA	F		
	GAATCCATTGCCATCCTT	R	~1700bp	~700bp
	AAGTAGAGGCAGCAACCA	F	~560bp	
AT3G25880	CACACTTGCACAATTCTC	F		
	TGAACTGTAAACCGGAAC	R	~2200bp	~1200bp
	CCACCAAGGACGAGATTC	F	~770bp	
AT4G10060	AGAGCCTGACCCTGAACT	F		
	ATGGCTCCACAACAACAA	R	~4000bp	~700bp
	TACCCTCCAACTGCGTGA	F	~580bp	
AT4G35165	TCCGTGAGTGTCGCTAAT	F		
	GAATACGCATCCCTGAGT	R	~2100bp	~1700bp
	GTCGTGCCTTCAAGTCTA	R	~1300bp	
AT5G42350	TCTGGAGCTGGTAATGAA	F		
	TCAGTGGCACCAACAATG	R	~2400bp	~1400bp
	GACTACATCAGCGCCTTA	R	~1100bp	
AT5G48175	TCTTCAGCGACAGCCGAT	F		
	GGCTAGTGAACAGTGGAA	R	~2300bp	~1200bp
	TGGAAACAGGATACTCAC	R	~250bp	
AT2G23985	GCCACAAGTATCGAACAT	F		
	CAGGGCCTTACTACTTCA	R	~3000bp	~1400bp or
	AACAAGGGCGATGAGGAT	F	~900bp	~2400bp
AT2G01360	CCTTGGAATTGCGTAGAA	F		
	CAGAGTTGGAGCATGGTT	R	~1700bp	~1000bp

	TGCTTACACTGCGCTGAT	F	~700bp	
AT4G29710	CGTAGTTCACCGTCATCA	F		
	GTTTGGGCTACATTCAGA	R	~1600bp	~600bp
	AAAGTCCACTGGGTCATA	R	~200bp	
AT5G65050	CCGTACCCGAGTCATATT	F		
	TCTAGGGTTCTTCACGCT	R	~4400bp	~400bp
	ACAGAAGCTCATGCGAAA	F	~160bp	
AT4G06490	TCCCGACTATGCTCCGTA	F		
	CTCAACGTGCATGGACTT	R	~1500bp	~800bp
	TTCCGAGCTGCCTATGTA	F	~450bp	
AT5G38670	GACGGTTGTGAGGACGAA	F		
	ACGTGTGAGACCGGCAAT	R	~2300bp	~800bp
	TCCGGTGAAGCTATGAGA	R	~500bp	
AT1G52440	CTAGGTCTGGTTGCACAT	F		
	AGGTGGAGACTGGAGCAT	R	~2700bp	~400bp or
	CGTCAAGTTCACACCATT	F	~800bp	~1200bp
AT2G29920	CAGGTCATTTCGGTCTAT	F		
	CTGTATTGCTGTTGCTGA	R	~2000bp	~900bp
	CCAATGGAAGGAACTGTT	R	~660bp	
AT5G26912	TCGACCACCGTTTAGTAT	F		
	GTTGGCATACCGAGATAA	R	~800bp	~360bp
	AACCCTCGACAACTAGAT	F	~200bp	
AT2G28426	GGACGTGAAATTCGGCAA	F		
	TGAACCTGCGATTGAGTA	R	~800bp	~500bp
	CAGCGAGATTCAACACAA	R	~350bp	
AT4G38280	CGCTCCTCCGATTGATTA	F		
	TGAACGCACCAGCAACTA	R	~2000bp	~800bp
	GCTGCTTTGGATGTGTCA	R	~300bp	
AT5G18880	GAAGGTCCAATCAGCATC	F		
	CGCTAGTCGATATGCAGA	R	~1900bp	~800bp
	ATCCGTCCAGGCATCATA	F	~550bp	
AT1G02590	CGATTCCGGTTAAGACTA	F		
	CGAACCATCACAGCTATT	R	~1400bp	~800bp
	GAAGATTGAAGCTGCTGC	R	~200bp	
AT3G11060	GTTATCGACAAGCTGCAA	F		
	CCTTCGTATCCGTTCAGT	R	~1800bp	~1000bp
	CGAAACGCTTGTCTGATT	R	~250bp	
AT2G24617	CGGTGTGCAAAGAGAAGT	F		
	GTGGTATGGGTTGCGAAT	R	~1000bp	~550bp
	AGGTGGTTGTACGAGGAA	R	~300bp	
AT5G18010	GGATCTGAAATTCTCGGT	F		
	AGGGCACGTCTTATCTCA	R	~1000bp	~800bp
	AGTTTGGGTTTGCTCATC	R	~500bp	
AT5G26270	GGTTATCGGTGACAGTGA	F		

	TGGATGGACTTATGGACA	R	~2600bp	~2000bp
	CTCTTGTGTTACGTTGGA	F	~800bp	
AT5G26950	GTCCTTCGCTGTCACACT	F		
	GTGCAGACAACCAAGATT	R	~2000bp	~1000bp
	GGATCTGGTCTGGGAAGT	R	~800bp	
AT1G59920	ACCACGCACTTCGAGATT	F		
	GCGTCTAAGCCAATTTCT	R	~1700bp	~700bp
AT1G43680	CCGATGGTTTTAGGTACT	F		
	GTAACAACTCCACCCTAT	R	~2100bp	~1100bp
	ACGTATTCACCTCTGCAT	F	~400bp	
AT2G34320	AATGATGTGTTGCGATAA	F		
	CCTACTCGGCCAATTCTA	R	~1800bp	~800bp
	CAGACGCTACATGGCAGT	F	~600bp	
AT5G56690	GTTATCCCGATTTGGCTA	F		
	TTGCTCCTCGCTGTAGTA	R	~2000bp	~680bp
	CGGGTATGATTCTGGTAT	F	~340bp	
AT1G05740	TCCAGTTGTCAATGCTCA	F		
	ACCCGACTTTGGACATAA	R	~1700bp	~780bp
	GCCAAGTTTCAGGTACAA	F	~380bp	
AT1G47497	TGACCCACCAATCCAGTA	F		
	GCGGCTATGTGAAGTGAT	R	~2200bp	~1250bp
	GGTTTCAGGAATGCAGAT	F	~800bp	
AT4G18540	GAATCGCAGTATCGTGAA	F		
	GTCCACTTGCACATCACA	R	~2400bp	~1000bp
	GAGACGTGTTCCTGTTGA	R	~580bp	
AT4G01535	TACAGCATGATCGGACTT	F		
	CCGGTGAAGAAGTTATGA	R	~2000bp	~900bp
	TACCAATGCTTCGGAGTT	F	~700bp	
AT1G48390	CTGAGATTTGCGGATGAT	F		
	TTTGCCTTGCTAGACGTA	R	~2600bp	~800bp
	AGCTTGACAATGGAATCT	F	~400bp	
AT4G09430	TGGCTAAGAATGCGGTAT	F		
	GCAGTGACTTGTCGCTAA	R	~6000bp	~900bp
	CACAATTTGGAAGCAGAA	F	~550bp	

A three-primer PCR amplification was used in our study. The first two primers are designed in 3’- and 5’-flanking regions and the last one is in the insertion of P/A genes. The present gene has two PCR products by the two pairs of primers while the absent gene has only one because of lacking the insertion region.
