# Supplementary material for: Variation of presence/absence genes among Arabidopsis populations
Source: BMC Evol Biol. 2012 Jun 14;12:86. doi: 10.1186/1471-2148-12-86 (PMC3433342; doi:10.1186/1471-2148-12-86)
Supplement: Additional file 3 — Figure S5.The P/A tree and accession tree. [file 1471-2148-12-86-S3.pdf]

A

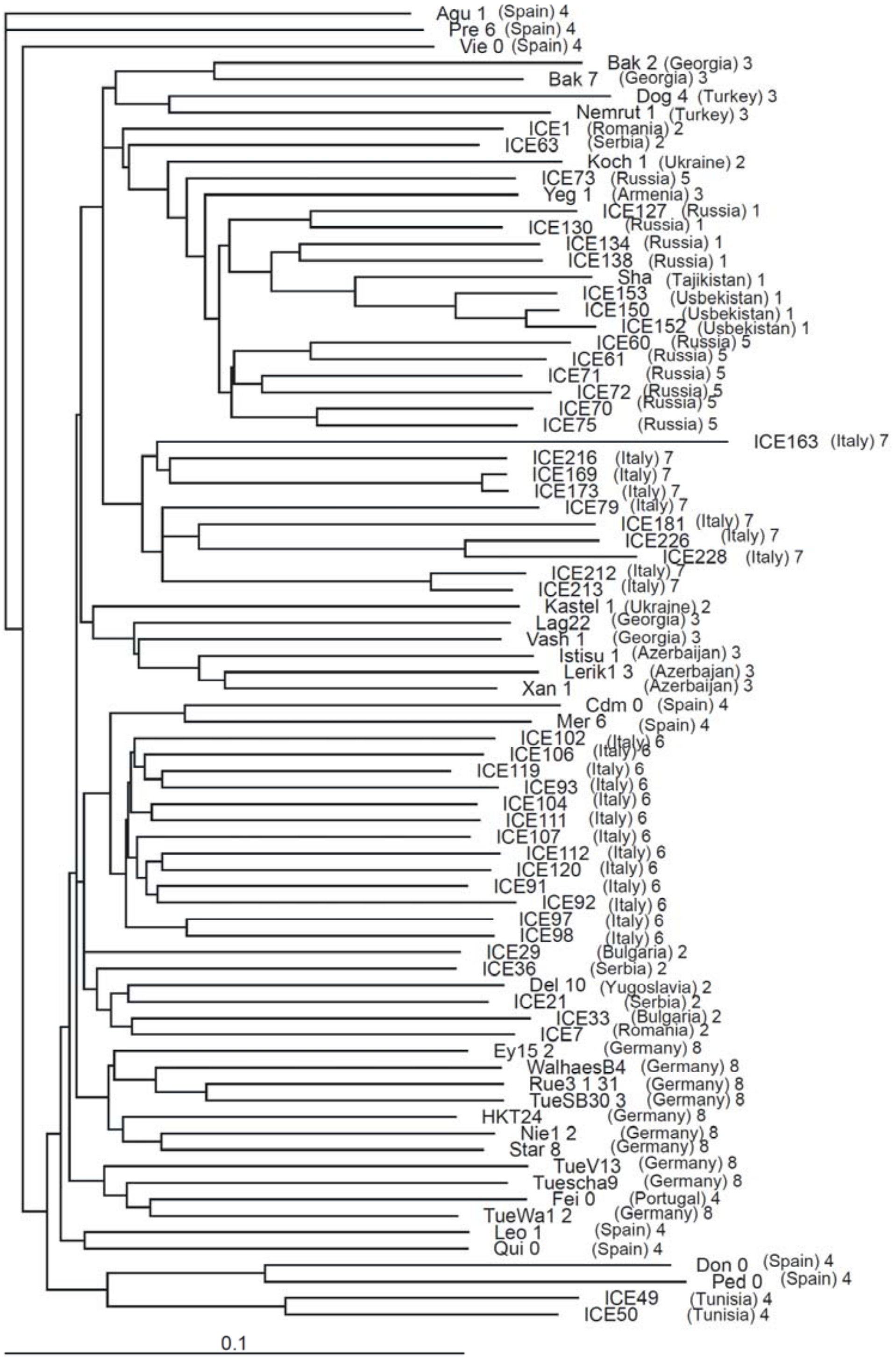
Agu 1 (Spain) 4
Pre 6 (Spain) 4
Vie 0 (Spain) 4
Bak 2 (Georgia) 3
Bak 7 (Georgia) 3
Dog 4 (Turkey) 3
Nemrut 1 (Turkey) 3
ICE1 (Romania) 2
ICE63 (Serbia) 2
Koch 1 (Ukraine) 2
ICE73 (Russia) 5
Yeg 1 (Armenia) 3
ICE127 (Russia) 1
ICE130 (Russia) 1
ICE134 (Russia) 1
ICE138 (Russia) 1
Sha (Tajikistan) 1
ICE153 (Usbekistan) 1
ICE150 (Usbekistan) 1
ICE152 (Usbekistan) 1
ICE60 (Russia) 5
ICE61 (Russia) 5
ICE71 (Russia) 5
ICE72 (Russia) 5
ICE70 (Russia) 5
ICE75 (Russia) 5
ICE163 (Italy) 7
ICE216 (Italy) 7
ICE169 (Italy) 7
ICE173 (Italy) 7
ICE79 (Italy) 7
ICE181 (Italy) 7
ICE226 (Italy) 7
ICE228 (Italy) 7
ICE212 (Italy) 7
ICE213 (Italy) 7
Kastel 1 (Ukraine) 2
Lag22 (Georgia) 3
Vash 1 (Georgia) 3
Istisu 1 (Azerbaijan) 3
Lerik1 3 (Azerbajan) 3
Xan 1 (Azerbaijan) 3
Cdm 0 (Spain) 4
Mer 6 (Spain) 4
ICE102 (Italy) 6
ICE106 (Italy) 6
ICE119 (Italy) 6
ICE93 (Italy) 6
ICE104 (Italy) 6
ICE111 (Italy) 6
ICE107 (Italy) 6
ICE112 (Italy) 6
ICE120 (Italy) 6
ICE91 (Italy) 6
ICE92 (Italy) 6
ICE97 (Italy) 6
ICE98 (Italy) 6
ICE29 (Bulgaria) 2
ICE36 (Serbia) 2
Del 10 (Yugoslavia) 2
ICE21 (Serbia) 2
ICE33 (Bulgaria) 2
ICE7 (Romania) 2
Ey15 2 (Germany) 8
WalhaesB4 (Germany) 8
Rue3 1 31 (Germany) 8
TueSB30 3 (Germany) 8
HKT24 (Germany) 8
Nie1 2 (Germany) 8
Star 8 (Germany) 8
TueV13 (Germany) 8
Tuescha9 (Germany) 8
Fei 0 (Portugal) 4
TueWa1 2 (Germany) 8
Leo 1 (Spain) 4
Qui 0 (Spain) 4
Don 0 (Spain) 4
Ped 0 (Spain) 4
ICE49 (Tunisia) 4
ICE50 (Tunisia) 4
0.1

B

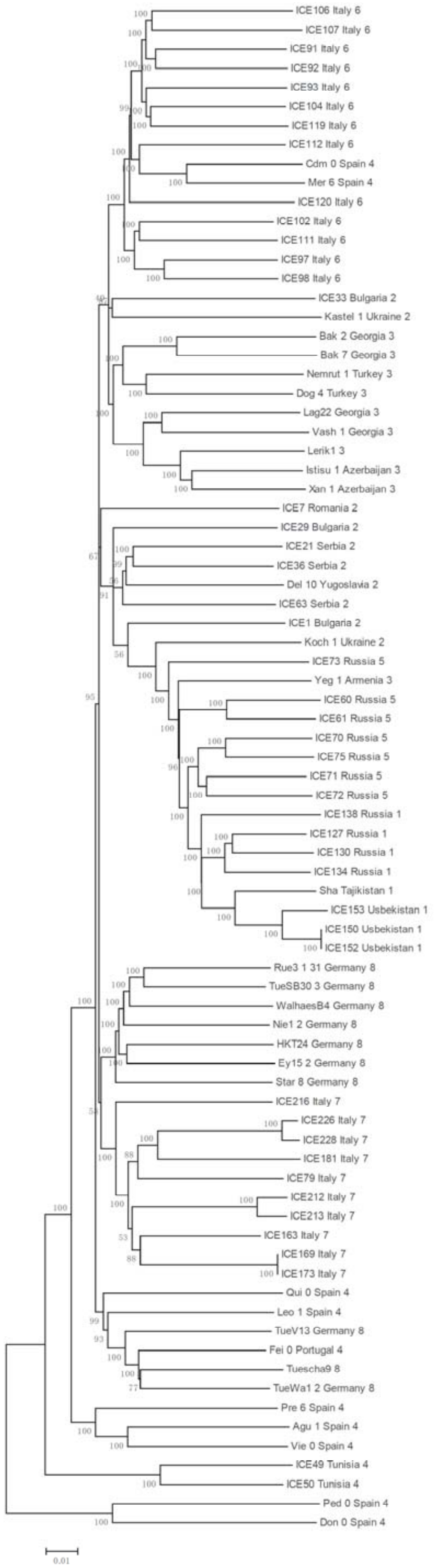
ICE106 Italy 6
ICE107 Italy 6
ICE91 Italy 6
ICE92 Italy 6
ICE93 Italy 6
ICE104 Italy 6
ICE119 Italy 6
ICE112 Italy 6
Cdm 0 Spain 4
Mer 6 Spain 4
ICE120 Italy 6
ICE102 Italy 6
ICE111 Italy 6
ICE97 Italy 6
ICE98 Italy 6
ICE33 Bulgaria 2
Kastel 1 Ukraine 2
Bak 2 Georgia 3
Bak 7 Georgia 3
Nemrut 1 Turkey 3
Dog 4 Turkey 3
Lag22 Georgia 3
Vash 1 Georgia 3
Lerik1 3
Istisu 1 Azerbaijan 3
Xan 1 Azerbaijan 3
ICE7 Romania 2
ICE29 Bulgaria 2
ICE21 Serbia 2
ICE36 Serbia 2
Del 10 Yugoslavia 2
ICE63 Serbia 2
ICE1 Bulgaria 2
Koch 1 Ukraine 2
ICE73 Russia 5
Yeg 1 Armenia 3
ICE60 Russia 5
ICE61 Russia 5
ICE70 Russia 5
ICE75 Russia 5
ICE71 Russia 5
ICE72 Russia 5
ICE138 Russia 1
ICE127 Russia 1
ICE130 Russia 1
ICE134 Russia 1
Sha Tajikistan 1
ICE153 Usbekistan 1
ICE150 Usbekistan 1
ICE152 Usbekistan 1
Rue3 1 31 Germany 8
TueSB30 3 Germany 8
WalhaesB4 Germany 8
Nie1 2 Germany 8
HKT24 Germany 8
Ey15 2 Germany 8
Star 8 Germany 8
ICE216 Italy 7
ICE226 Italy 7
ICE228 Italy 7
ICE181 Italy 7
ICE79 Italy 7
ICE212 Italy 7
ICE213 Italy 7
ICE163 Italy 7
ICE169 Italy 7
ICE173 Italy 7
Qui 0 Spain 4
Leo 1 Spain 4
TueV13 Germany 8
Fei 0 Portugal 4
Tuescha9 8
TueWa1 2 Germany 8
Pre 6 Spain 4
Agu 1 Spain 4
Vie 0 Spain 4
ICE49 Tunisia 4
ICE50 Tunisia 4
Ped 0 Spain 4
Don 0 Spain 4
0.01

Figure S5. The phylogenetic trees. (A) Accessions grouped relative to P/A frequencies. The phylogenetic tree was created based on neighbor-joining method with p-distance model by PAUP* 4.0. (B) The accession tree was constructed by using all SNPs of non-P/A genes among genomes, based on the Neighbor-joining method. The 80 accessions could be generally divided into 8 clades, representing the eight geographic regions where they were sampled. The numbers after each accessions were: (1) Central Asia; (2) East Europe; (3) Caukasus; (4) N-Africa and Spain; (5) Russia; (6) Southern Italy; (7) Southern Tyrol and (8) Tuebingen. The locational distribution could be found on 1001genomes website.
