# Supplementary material for: Variation of presence/absence genes among Arabidopsis populations
Source: BMC Evol Biol. 2012 Jun 14;12:86. doi: 10.1186/1471-2148-12-86 (PMC3433342; doi:10.1186/1471-2148-12-86)
Supplement: Additional file 4 — Table S1.The 80 accessions used in this study. [file 1471-2148-12-86-S4.pdf]

Table S1. The 80 accessions used in this study.

Name	NASC code	Alternative name	Latitude	Longitude	Country
Agu-1	CS76409	Agu-1	41.32	-1.3414	Spain
Bak-2	CS76392	Bak-2	41.7942	43.4767	Georgia
Bak-7	CS76393	Bak-7	41.7942	43.4767	Georgia
Cdm-0	CS76410	Cdm-0	39.7255	-5.7441	Spain
Del-10	CS76397	Del-10	44.9444	21.1828	Yugoslavia
Dog-4	CS76386	Dog-4	38.3011	42.2239	Turkey
Don-0	CS76411	Don-0	36.8323	-6.3598	Spain
Ey15-2	CS76399	Ey1.5-2	48.4345	8.7678	Germany
Fei-0	CS76412	Fei-0	40.9233	-8.5421	Portugal
HKT2.4	CS76404	HKT2-4	48.136	9.4033	Germany
ICE1	CS76373	Bolin-1	44.4604	25.7356	Romania
ICE102	CS76423	Galdo-1	40.5711	15.3215	Italy
ICE104	CS76367	Lago-1	39.1776	16.2601	Italy
ICE106	CS76365	Mammo-1	38.3623	16.2304	Italy
ICE107	CS76364	Mammo-2	38.3782	16.2174	Italy
ICE111	CS76361	Monte-1	40.2803	15.655	Italy
ICE112	CS76363	Moran-1	39.8315	16.1726	Italy
ICE119	CS76424	Timpo-1	39.2729	16.2677	Italy
ICE120	CS76425	Valsi-1	40.1763	16.4533	Italy
ICE127	CS76385	Kly1	51.3391	82.5724	Russia
ICE130	CS76384	Kly4	51.3184	82.5528	Russia
ICE134	CS76383	Koz2	51.3284	82.185	Russia
ICE138	CS76426	Leb-3	51.655	80.8151	Russia
ICE150	CS76379	Sij1	41.45	70.05	Usbekistan
ICE152	CS76380	Sij2	41.45	70.05	Usbekistan
ICE153	CS76381	Sij4	41.45	70.05	Usbekistan
ICE163	CS76353	Altenb-2	46.3716	11.2376	Italy
ICE169	CS76357	Bozen-1	46.513	11.331	Italy
ICE173	CS76358	Bozen-1	46.513	11.331	Italy
ICE181	CS76354	Mitterberg-1	46.3646	11.2835	Italy
ICE21	CS76370	Petro-1	44.3386	21.4603	Serbia
ICE212	CS76355	Castelfed-4	46.3378	11.2928	Italy
ICE213	CS76356	Castelfed-4	46.3378	11.2928	Italy
ICE216	CS76351	Rovero-1	46.2543	11.167	Italy
ICE226	CS76349	Vezzano-2	46.6295	10.8161	Italy
ICE228	CS76350	Vezzano-2	46.6295	10.8161	Italy
ICE29	CS76419	Slavi-1	41.4275	23.647	Bulgaria
ICE33	CS76372	Jablo-1	41.588	25.1988	Bulgaria
ICE36	CS76369	Dobra-1	44.8373	20.1566	Serbia
ICE49	CS76347	Aitba-2	31.484	-7.4499	Tunisia

ICE50	CS76348	Toufl-1	31.4687	-7.4166	Tunisia
ICE60	CS76377	Stepn-2	54.0857	60.4609	Russia
ICE61	CS76378	Stepn-1	54.0599	60.4789	Russia
ICE63	CS76420	Copac-1	46.1088	21.9523	Serbia
ICE7	CS76371	Lecho-1	41.428	23.4993	Romania
ICE70	CS76421	Borsk-2	50.0389	51.7457	Russia
ICE71	CS76375	Shigu-1	53.3328	49.4807	Russia
ICE72	CS76374	Shigu-2	53.332	49.4804	Russia
ICE73	CS76376	Kidr-1	51.3071	57.5612	Russia
ICE75	CS76422	Krazo-2	53.088	52.0001	Russia
ICE79	CS76352	Voeran-1	46.36	11.23	Italy
ICE91	CS76362	Angel-1	38.6191	16.1678	Italy
ICE92	CS76366	Angit-1	38.7631	16.2416	Italy
ICE93	CS76368	Apost-1	39.0085	16.4678	Italy
ICE97	CS76359	Ciste-1	41.6156	12.8687	Italy
ICE98	CS76360	Ciste-2	41.6156	12.8687	Italy
Istisu-1	CS76389	Istisu-1	38.9786	48.5594	Azerbaijan
Kastel-1	CS76395	Kastel-1	44.6419	34.3814	Ukraine
Koch-1	CS76396	Koch-1	50.3553	29.3244	Ukraine
Lag2.2	CS76390	Lag2-2	41.8296	46.2831	Georgia
Leo-1	CS76413	Leo-1	41.7959	-3.1147	Spain
Lerik1-3	CS76388	Lerik1-3	38.7406	48.6131	Azerbaijan
Mer-6	CS76414	Mer-6	38.9159	-6.3376	Spain
Nemrut-1	CS76398	Nemrut-1	38.6425	42.2394	Turkey
Nie1-2	CS76402	Nie1-2	48.5179	8.8026	Germany
Ped-0	CS76415	Ped-0	40.7385	-3.8978	Spain
Pre-6	CS76416	Pra-6	41.0504	-3.5395	Spain
Qui-0	CS76417	Qui-0	42.6918	-6.9301	Spain
Rue3-1-31	CS76406	Ru3.1-31	48.564	9.1595	Germany
Sha	CS76382	Sha	38.35	68.48	Tajikistan
Star-8	CS76400	Star-8	48.4345	8.8167	Germany
TueSB30-3	CS76403	Tu-SB30-3	48.5334	9.0583	Germany
Tuescha9	CS76401	Tu-Scha-9	48.5344	9.0503	Germany
TueV13	CS76407	Tu-V-13	48.5232	9.052	Germany
TueWa1-2	CS76405	Tu-Wa1-2	48.5342	9.0345	Germany
Vash-1	CS76391	Vash-1	41.2381	46.3728	Georgia
Vie-0	CS76418	Vie-0	42.6266	0.7626	Spain
WalhaesB4	CS76408	Wal-HasB-4	48.5956	9.1855	Germany
Xan-1	CS76387	Xan-1	38.6536	48.7992	Azerbaijan
Yeg-1	CS76394	Yeg-1	39.8692	45.3622	Armenia
