# Supplementary material for: Variation of presence/absence genes among Arabidopsis populations
Source: BMC Evol Biol. 2012 Jun 14;12:86. doi: 10.1186/1471-2148-12-86 (PMC3433342; doi:10.1186/1471-2148-12-86)
Supplement: Additional file 5 — Figure S1.Distributions of P/A genes on chromosomes. [file 1471-2148-12-86-S5.pdf]

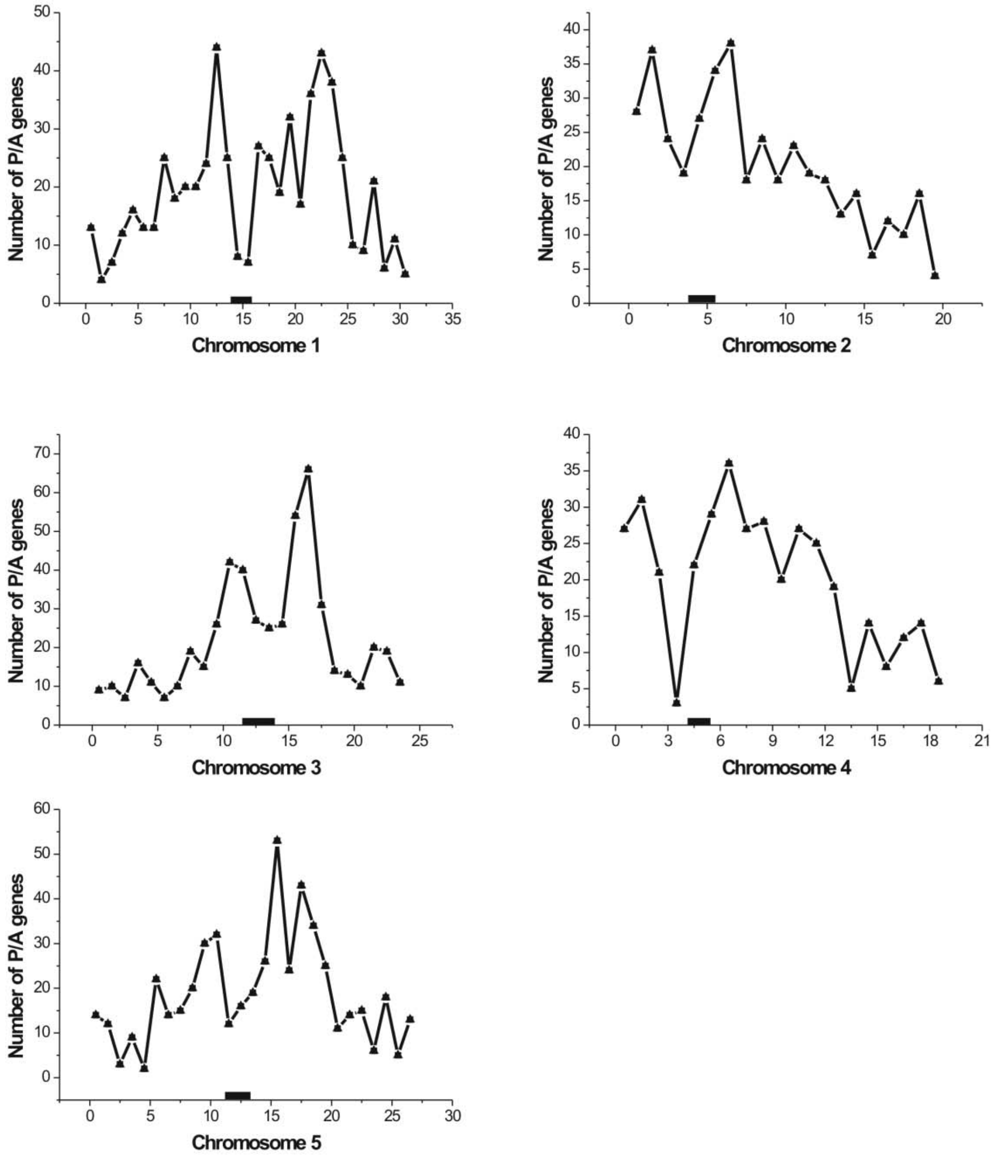

Figure S1. Distributions of P/A genes on chromosomes. The x-axis represents the physical distance (Mb) on the chromosome. The y-axis represents the number of P/A genes for each Mb of chromosomal length. The thick black bar represents the centromeric region.
