# Supplementary material for: Variation of presence/absence genes among Arabidopsis populations
Source: BMC Evol Biol. 2012 Jun 14;12:86. doi: 10.1186/1471-2148-12-86 (PMC3433342; doi:10.1186/1471-2148-12-86)
Supplement: Additional file 6 — Table S3.Distributions of P/A genes on chromosomes. [file 1471-2148-12-86-S6.pdf]

Table S3. Distributions of P/A genes on chromosomes.

Chromosome	D(Mb)	N_{genes}	$N_{P/A}$	$P_{P/A}$	N_c	P_c
Chr 1	0.5	299	13	0.0435	5	0.385
	1.5	280	4	0.0143	0	0
	2.5	289	7	0.0242	3	0.429
	3.5	281	12	0.0427	0	0
	4.5	290	16	0.0552	8	0.5
	5.5	298	13	0.0436	8	0.615
	6.5	272	13	0.0478	2	0.154
	7.5	250	25	0.1	13	0.52
	8.5	272	18	0.0662	10	0.556
	9.5	253	20	0.0791	9	0.45
	10.5	229	20	0.0873	12	0.6
	11.5	216	24	0.1111	7	0.292
	12.5	183	44	0.2404	12	0.273
	13.5	78	25	0.3205	4	0.16
	14.5	21	8	0.381	0	0
	15.5	17	7	0.4118	0	0
	16.5	103	27	0.2621	4	0.148
	17.5	203	25	0.1232	8	0.32
	18.5	243	19	0.0782	4	0.211
	19.5	258	32	0.124	7	0.219
	20.5	273	17	0.0623	6	0.353
	21.5	203	36	0.1773	15	0.417
	22.5	247	43	0.1741	27	0.628
	23.5	249	38	0.1526	23	0.605
	24.5	251	25	0.0996	11	0.44
	25.5	232	10	0.0431	2	0.2
	26.5	264	9	0.0341	2	0.222
	27.5	285	21	0.0737	7	0.333
	28.5	268	6	0.0224	2	0.333
	29.5	260	11	0.0423	1	0.091
	30.5	132	5	0.0379	0	0
Chr 2	0.5	223	28	0.126	9	0.321
	1.5	185	37	0.2	14	0.378
	2.5	74	24	0.324	4	0.167
	3.5	83	19	0.229	0	0
	4.5	44	27	0.614	3	0.111
	5.5	77	34	0.442	6	0.176
	6.5	137	38	0.277	7	0.184
	7.5	235	18	0.077	6	0.333
	8.5	269	24	0.089	6	0.25

	9.5	268	18	0.067	5	0.278
	10.5	234	23	0.098	6	0.261
	11.5	244	19	0.078	8	0.421
	12.5	242	18	0.074	6	0.333
	13.5	277	13	0.047	5	0.385
	14.5	289	16	0.055	3	0.188
	15.5	271	7	0.026	2	0.286
	16.5	277	12	0.043	3	0.25
	17.5	273	10	0.037	4	0.4
	18.5	307	16	0.052	5	0.313
	19.5	199	4	0.02	2	0.5
Chr 3	0.5	329	9	0.027	2	0.04
	1.5	296	10	0.034	1	0.014
	2.5	297	7	0.024	2	0.024
	3.5	308	16	0.052	5	0.039
	4.5	267	11	0.041	6	0.06
	5.5	294	7	0.024	2	0.044
	6.5	274	10	0.036	1	0.047
	7.5	270	19	0.07	3	0.093
	8.5	236	15	0.064	5	0.076
	9.5	263	26	0.099	12	0.076
	10.5	210	42	0.2	14	0.095
	11.5	107	40	0.374	4	0.224
	12.5	33	27	0.818	1	1.333
	13.5	28	25	0.893	9	0.893
	14.5	44	26	0.591	0	0.182
	15.5	116	54	0.466	6	0.06
	16.5	203	66	0.325	20	0.133
	17.5	238	31	0.13	13	0.105
	18.5	277	14	0.051	5	0.069
	19.5	306	13	0.042	3	0.105
	20.5	281	10	0.036	4	0.06
	21.5	291	20	0.069	11	0.124
	22.5	276	19	0.069	4	0.156
	23.5	152	11	0.072	3	0.25
Chr 4	0.5	264	27	0.102	14	0.519
	1.5	173	31	0.179	6	0.194
	2.5	136	21	0.154	3	0.143
	3.5	9	3	0.333	1	0.333
	4.5	31	22	0.71	0	0
	5.5	128	29	0.227	5	0.172
	6.5	224	36	0.161	11	0.306
	7.5	239	27	0.113	10	0.37

	8.5	256	28	0.109	16	0.571
	9.5	249	20	0.08	11	0.55
	10.5	256	27	0.105	8	0.296
	11.5	268	25	0.093	10	0.4
	12.5	275	19	0.069	9	0.474
	13.5	303	5	0.017	2	0.4
	14.5	277	14	0.051	4	0.286
	15.5	265	8	0.03	2	0.25
	16.5	299	12	0.04	3	0.25
	17.5	269	14	0.052	3	0.214
	18.5	176	6	0.034	1	0.167
Chr 5	0.5	296	14	0.047	6	0.429
	1.5	291	12	0.041	4	0.333
	2.5	291	3	0.01	0	0
	3.5	279	9	0.032	3	0.333
	4.5	272	2	0.007	1	0.5
	5.5	291	22	0.076	8	0.364
	6.5	276	14	0.051	7	0.5
	7.5	246	15	0.061	1	0.067
	8.5	221	20	0.09	8	0.4
	9.5	204	30	0.147	9	0.3
	10.5	88	32	0.364	7	0.219
	11.5	18	12	0.667	0	0
	12.5	26	16	0.615	0	0
	13.5	86	19	0.221	5	0.263
	14.5	172	26	0.151	8	0.308
	15.5	233	53	0.227	37	0.698
	16.5	276	24	0.087	10	0.417
	17.5	249	43	0.173	15	0.349
	18.5	234	34	0.145	11	0.324
	19.5	281	25	0.089	9	0.36
	20.5	256	11	0.043	1	0.091
	21.5	272	14	0.051	4	0.286
	22.5	289	15	0.052	6	0.4
	23.5	285	6	0.021	1	0.167
	24.5	281	18	0.064	12	0.667
	25.5	307	5	0.016	1	0.2
	26.5	284	13	0.046	4	0.308

D represents the physical distance (Mb) on the chromosome. In this column, the number is the central position of this chromosomal section. N_{genes} represents the total gene number for each 1 Mb section. $N_{P/A}$ and $P_{P/A}$ represent the number and

proportion of P/A genes in this section. N_c is the number of clustered P/A genes and P_c is the proportion, equal to N_c divided by $N_{P/A}$.
