# Supplementary material for: Variation of presence/absence genes among Arabidopsis populations
Source: BMC Evol Biol. 2012 Jun 14;12:86. doi: 10.1186/1471-2148-12-86 (PMC3433342; doi:10.1186/1471-2148-12-86)
Supplement: Additional file 7 — Figure S3.GO categories of P/A genes. [file 1471-2148-12-86-S7.pdf]

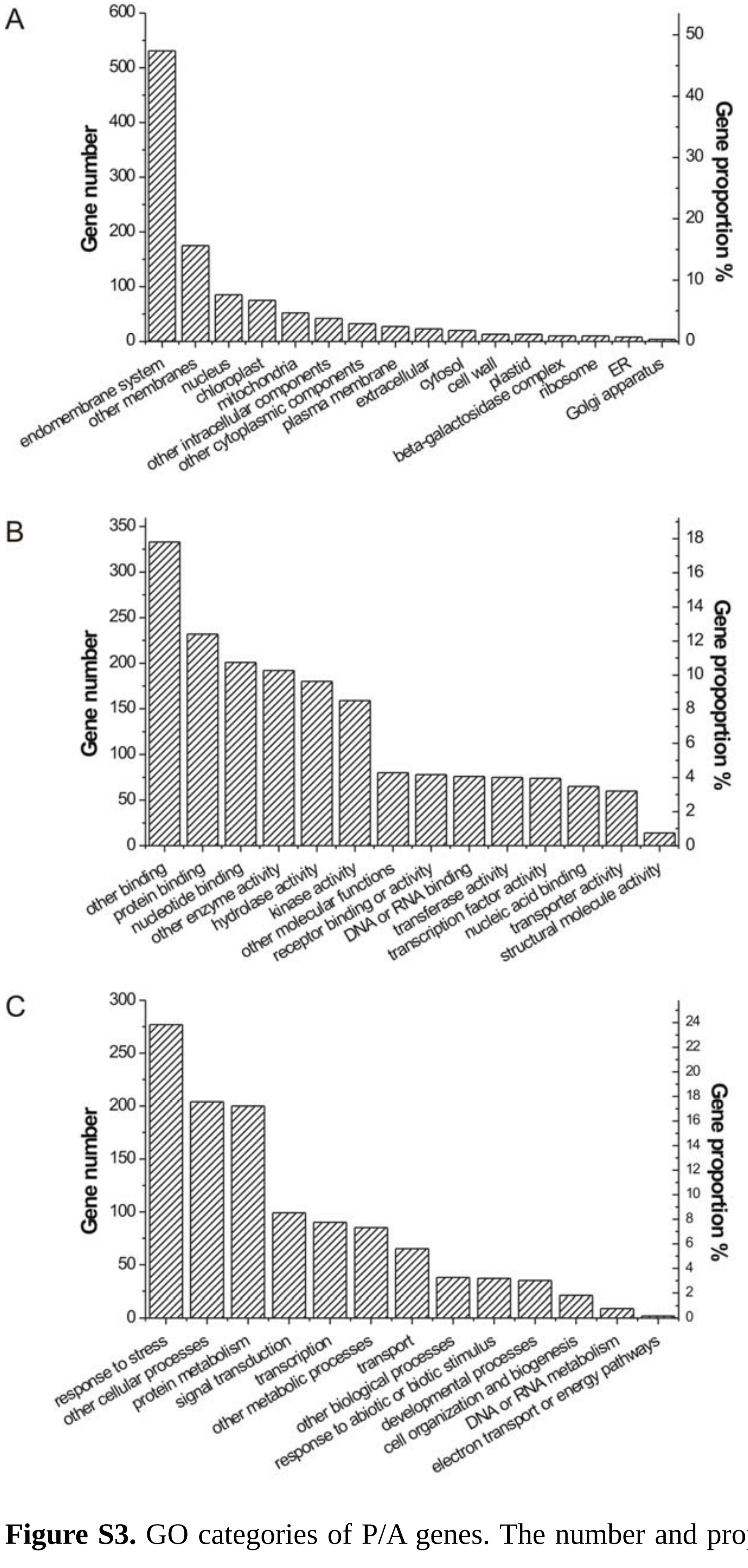


**Figure S3.** GO categories of P/A genes. The number and proportion of P/A genes in three categories, cellular component (A), molecular function (B) and biological

process (C).
