# Supplementary material for: Variation of presence/absence genes among Arabidopsis populations
Source: BMC Evol Biol. 2012 Jun 14;12:86. doi: 10.1186/1471-2148-12-86 (PMC3433342; doi:10.1186/1471-2148-12-86)
Supplement: Additional file 9 — Figure S4.Co-variation between absent allele frequency and nucleotide diversity or gene length. [file 1471-2148-12-86-S9.pdf]

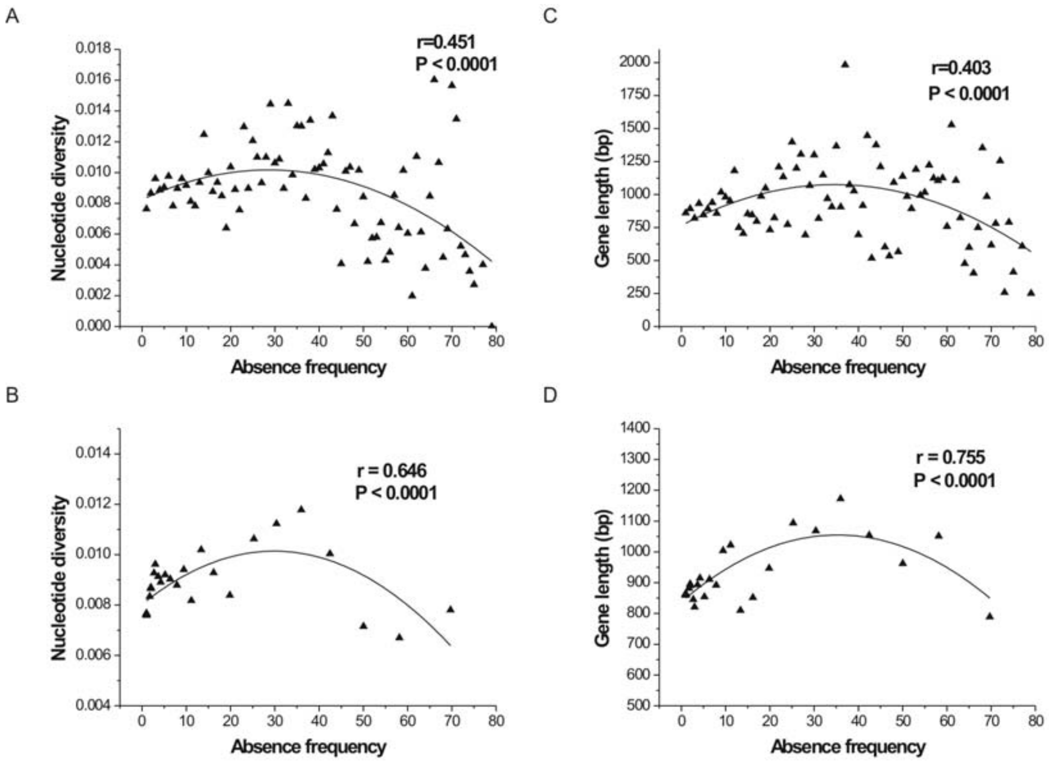


**Figure S4.** Co-variation between absent allele frequency and nucleotide diversity (A and B) or gene length (C and D). (A and C) there are 79 dots in both figures, from 1 to 79 on x-axis and each dot represents the average value within one absent allele frequency. (B and D) genes within the same absent frequency were randomly sorted and then the 2407 genes were splitted into 29 bins and each bin contains 83 genes.
